# Automated light-induced synthesis of ^89^Zr-radiolabeled antibodies for immuno-positron emission tomography

**DOI:** 10.1038/s41598-021-04626-5

**Published:** 2022-01-13

**Authors:** Simon Klingler, Jason P. Holland

**Affiliations:** Department of Chemistry, University of Zurich, Winterthurerstrasse 190, 8057 Zurich, Switzerland

**Keywords:** Photochemistry, Bioinorganic chemistry, Chemistry

## Abstract

Clinical production of ^89^Zr-radiolabeled antibodies (^89^Zr-mAbs) for positron emission tomography imaging relies on the pre-conjugation of desferrioxamine B (DFO) to the purified protein, followed by isolation and characterization of the functionalized intermediate, and then manual radiosynthesis. Although highly successful, this route exposes radiochemists to a potentially large radiation dose and entails several technological and economic hurdles that limit access of ^89^Zr-mAbs to just a specialist few Nuclear Medicine facilities worldwide. Here, we introduce a fully automated synthesis box that can produce individual doses of ^89^Zr-mAbs formulated in sterile solution in < 25 min starting from [^89^Zr(C_2_O_4_)_4_]^4–^ (^89^Zr-oxalate), our good laboratory practice-compliant photoactivatable desferrioxamine-based chelate (DFO-PEG_3_-ArN_3_), and clinical-grade antibodies without the need for pre-purification of protein. The automated steps include neutralization of the ^89^Zr-oxalate stock, chelate radiolabeling, and light-induced protein conjugation, followed by ^89^Zr-mAb purification, formulation, and sterile filtration. As proof-of-principle, ^89^ZrDFO-PEG_3_-azepin-trastuzumab was synthesized directly from Herceptin in < 25 min with an overall decay-corrected radiochemical yield of 20.1 ± 2.4% (*n* = 3), a radiochemical purity > 99%, and chemical purity > 99%. The synthesis unit can also produce ^89^Zr-mAbs via the conventional radiolabeling routes from pre-functionalized DFO-mAbs that are currently used in the clinic. This automated method will improve access to state-of-the-art ^89^Zr-mAbs at the many Nuclear Medicine and research institutions that require automated devices for radiotracer production.

## Introduction

Automation is common practice in the synthesis of clinical-grade radiopharmaceuticals. For example, the small-molecule, metal-based radiotracers ^68^Ga-PSMA-11 and ^177^Lu-PSMA-617 are typically produced by automated synthesis modules that perform both the radiolabeling and purification steps^1–4^. In these examples, the chemical reaction is a one-step process (metal ion complexation) and efficient radiolabeling produces the isolated and fully formulated radiopharmaceutical with high decay-corrected radiochemical yields (RCYs > 80%) and radiochemical purity (RCP > 95%). It is important to note that the metalation step typically runs to full decay-corrected radiochemical conversion (RCC > 95%). Activity losses stem from inefficient transfer along fluid pathways or imperfect recovery during the purification step. More complex chemical reactions or multiple synthetic steps can also be automated but these processes are usually encountered only in the production of small-molecule ^11^C- and ^18^F-labeled PET tracers^5–7^. For instance, ^11^C-radiochemistry often requires the use of non-aqueous solvents and the synthesis of reactive intermediates, such as ^11^C-methyl iodide, before subsequent reactions with a precursor give the desired radiotracer. In ^11^C- and ^18^F-radiochemistry, the labeling precursors often contain protected functional groups that are unmasked by deprotection steps following the introduction of the radionuclide^8–11^. This chemical flexibility facilitates the ‘*Total Radiosynthesis*’ of complex drug molecules where the radionuclide is incorporated in positions that are simply not accessible using the more common late-stage chemistry^9,12^.

In the clinic, automation improves the reproducibility of radiotracer synthesis, and helps minimize radiation exposure to radiochemists who frequently operate with GBq amounts of radioactivity^13^. Most automatic radiosynthesizers are placed inside (mini)hot cells and are operated remotely via a computer interface. Radiotracer automation also improves traceability, documentation management and compliance with regulatory requirements for quality control^14^.

There are two prominent examples for the automated synthesis of ^89^Zr-labeled monoclonal antibodies (^89^Zr-mAbs)^3,15^. In both reports, the radiolabeling reaction relies on the use of a pre-functionalized desferrioxamine-antibody conjugate (DFO-mAb). In 2016, Wright et al*.* performed the ^89^Zr-radiolabeling of DFO-Bz-NCS-trastuzumab on a microfluidic chip followed by manual purification. The procedure was performed in 45–60 min and gave an isolated product with sufficient activity yield for multiple patient doses^15^. More recently, Poot et al*.* reported the fully automated radiolabeling and purification of ^89^Zr-mAbs in 2019^3^. In this example, single patient doses of purified ^89^ZrDFO-*N*-Succ-rituximab and ^89^ZrDFO-*N*-Succ-cetuximab were produced in 77 min starting from ^89^Zr-oxalate and the respective pre-functionalized DFO-mAb^3^. The DFO-mAb radiolabeling precursors are produced by DFO-conjugation to lysine residues on the protein using reagents bearing the activated ester *N*-hydroxysuccinimide (NHS) or a benzylisothiocyanate (Bz-NCS) group which form amide and thiourea bonds, respectively^16,17^. DFO-mAb conjugates are typically prepared in advance, characterized, stored, and radiolabeled on demand, and so far, this holds true for all automated and manual production of ^89^Zr-mAbs. In general, this chemistry is extremely successful and has facilitated clinical translation of many ^89^Zr-mAbs^18–22^. However, the necessity to produce, characterize and store the DFO-mAb intermediate presents several technological and financial limitations—synthesizing sufficient material for toxicological studies is both difficult and expensive—that mean accessing ^89^Zr-mAbs is not always feasible in smaller nuclear medicine facilities. In addition, the long-term storage of an intermediate can lead to questions over the stability and shelf-life of the material.

**Figure 1:**
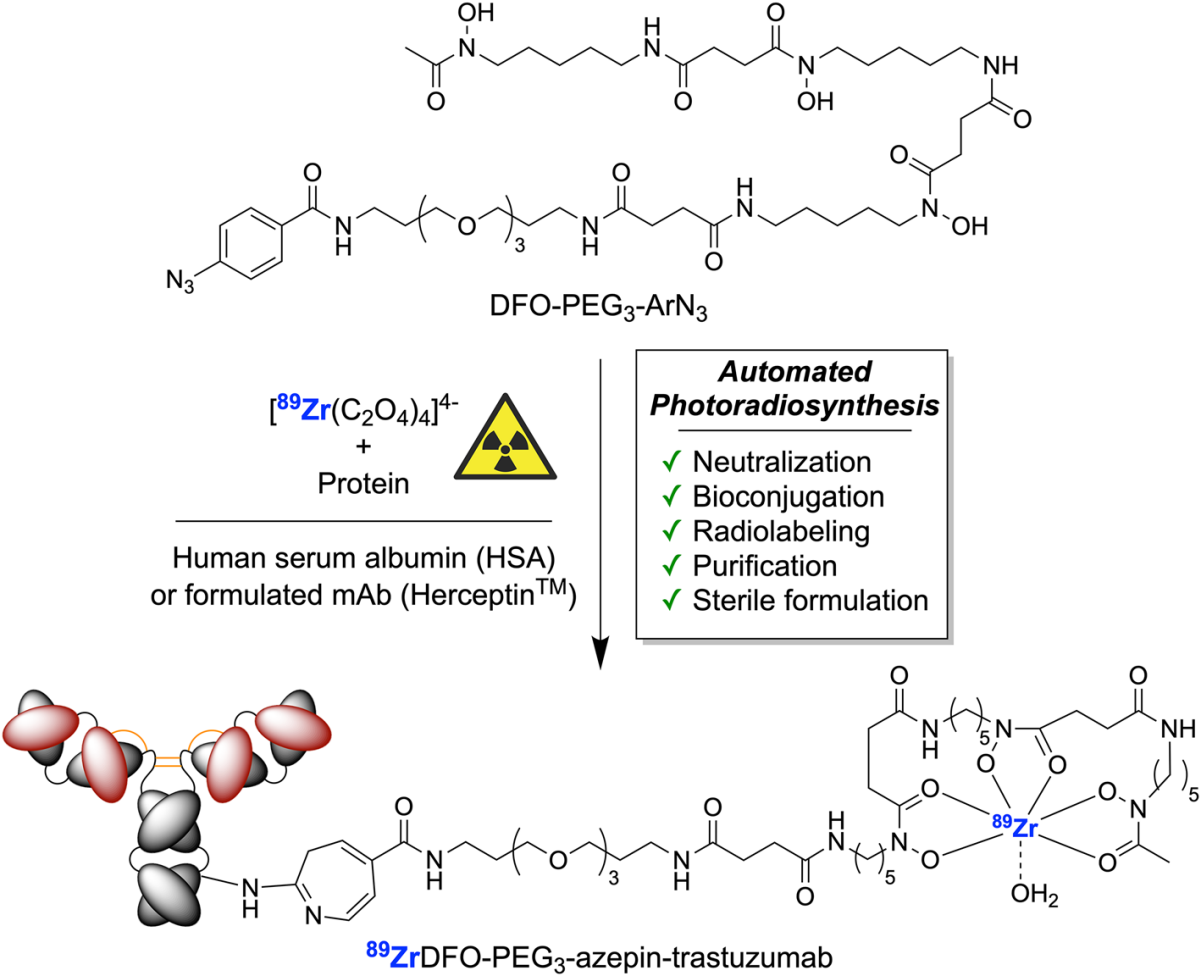
Overview of the multi-component, one-pot reaction for the photoradiosynthesis of ^89^Zr-radiolabeled proteins. The production of ^89^Zr-mAbs with ALISI involves automated neutralization of the oxalic acid in the ^89^Zr-stock solution, chelate radiolabeling, light-induced protein conjguation, in-line purification, sterile filtration and product formulation in < 25 min at the push of a button.

Recently, we developed a one-pot route that combines the bioconjugation and radiolabeling steps using new DFO-chelates bearing a photoactivatable aryl azide group (ArN_3_)^23–26^. Three key features of this alternative photoradiochemical method for making ^89^Zr-mAbs are: *i*) rapid reaction times that depend on the rate-limiting photoactivation step; *ii*) high chemical tolerance of the light-induced bioconjugation process with many components of mAb formulation buffers, and *iii*) avoidance of the need to use pre-functionalized DFO-mAbs. The compatibility of the photolabeling process with common mAb formulation buffers, including high concentrations amino acids (e.g. histidine), surfactants (polysorbate-80), sugars (α,α-trehalose), antioxidants (ascorbate), and various salts such as phosphate buffered saline (PBS) mean that the chemistry often works without the need to pre-purify the protein from clinical-grade stocks. This critical difference between our photoradiosynthesis approach and the classic multiple step routes for making ^89^Zr-mAbs led us to postulate that it could be feasible to make an automated radiosynthesizer that combines all critical steps required in the manufacture of ^89^Zr-mAbs, namely, oxalic acid neutralization, bioconjugation, radiolabeling, purification and sterile formulation (Fig. 1).

**Figure 2:**
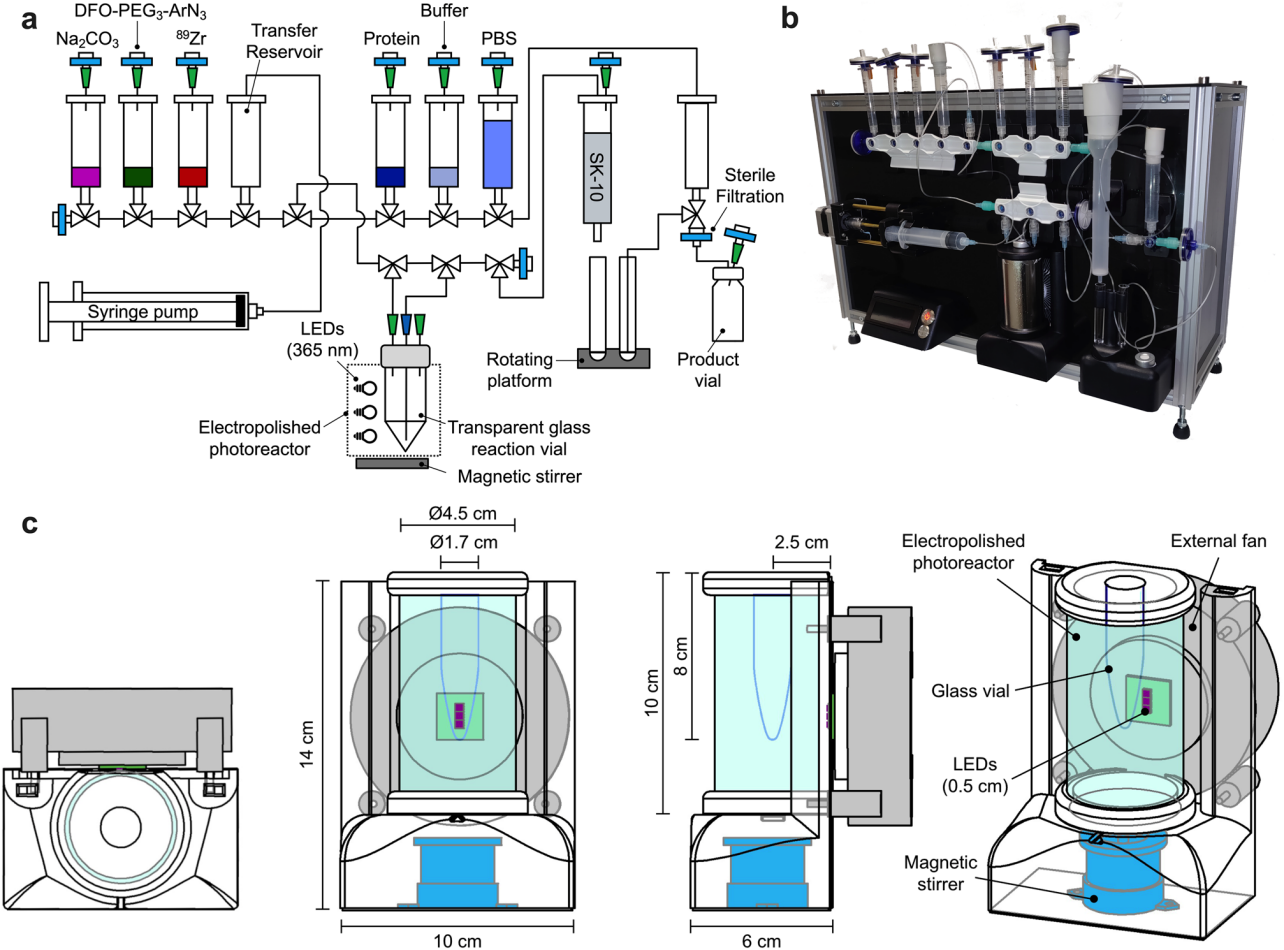
Design and construction of the automated radiosynthesizer unit. (**a**) Schematic representation of the ALISI plumbing diagram and the experimental setup used for the automated photoradiosynthesis and purification of ^89^Zr-mAbs. (**b**) Photograph of our custom-built ALISI device. (**c**) Technical drawing of the photoreactor design showing (from left to right) the top, front, side, and 3-dimensional view.

Here, we report the design, manufacture, and proof-of-concept pre-clinical evaluation of ALISI – a prototype radiosynthesizer for the fully automated light-induced synthesis of ^89^Zr-mAbs for immuno-positron emission tomography (PET). An overview of the design and plumbing diagram of the ALISI system is presented in Fig. 2 with full descriptions given in the Methods section.

## Results

**Figure 3:**
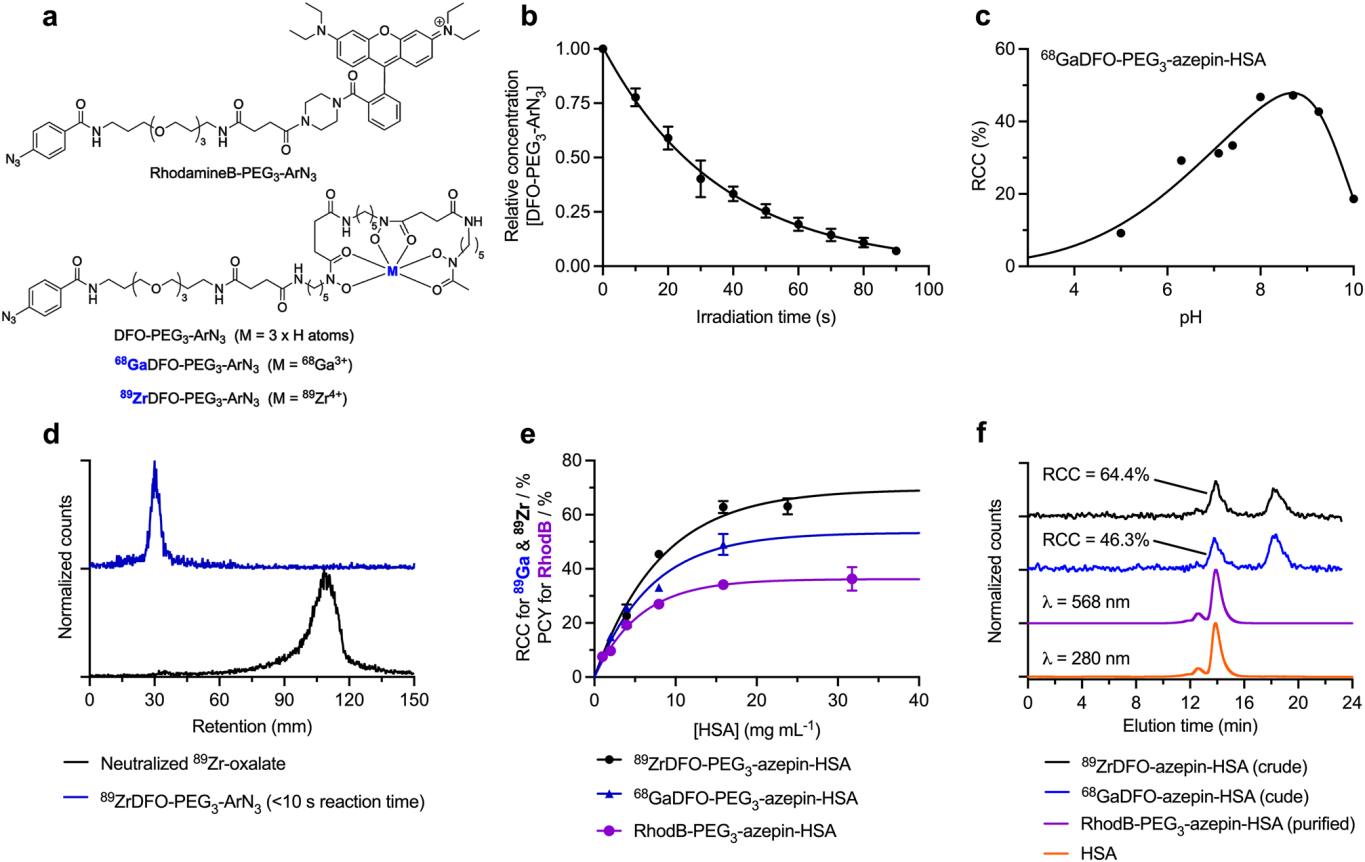
Experimental optimization of the automated photo(radio)synthesis of labeled protein. (**a**) Chemical structures of RhodB-PEG_3_-ArN_3_ and various DFO-PEG_3_-ArN_3_ compounds used in model reactions. (**b**) Photoactivation kinetics indicating the change in DFO-PEG_3_-ArN_3_ concentration over time measured by peak integration of HPLC chromatograms. (**c**) A plot of the radiochemical conversion (RCC) *versus* pH for the synthesis of ^68^GaDFO-PEG_3_-azepin-HSA. (**d**) Radio-ITLC traces (DTPA eluent) of neutralized ^89^Zr-oxalate before (black) and immediately after (blue) the addition of the photoactivatable DFO-PEG_3_-ArN_3_ chelate indicating rapid complexation. (**e**) Data on the experimental radiochemical conversion (RCC/%) or photochemical conversion yields (PCY/%) *versus* protein concentration for model reactions between HSA and RhodB-PEG_3_-ArN_3_ (pink), pre-labeled ^68^GaDFO-PEG_3_-ArN_3_ (blue), and simultaneous photoradiolabeling with ^89^Zr-oxalate plus DFO-PEG_3_-ArN_3_ (black). (**f**) Representative SEC-HPLC chromatograms of HSA (orange), purified RhodB-PEG_3_-azepin-HSA (pink) produced by full automation, and crude reaction mixtures (pre-purification) showing the light-induced formation of ^68^GaDFO-PEG_3_-azepin-HSA (blue) and ^89^ZrDFO-PEG_3_-azepin-HSA (black) at [HSA] = 15.9 mg mL^-1^.

Prior to developing the fully automated synthesis of ^89^Zr-mAbs, we first tested different aspects of the ALISI system by using model reactions involving the bioconjugation between human serum albumin (HSA) and three photoactivatable compounds. The compounds used were RhodB-PEG_3_-ArN_3_, pre-radiolabeled ^68^GaDFO-PEG_3_-ArN_3_, and the free ligand (DFO-PEG_3_-ArN_3_) together with ^89^Zr-oxalate which forms ^89^ZrDFO-PEG_3_-ArN_3_ in situ. Chemical structures are shown in Figs. 1 and 3a, and reaction details are presented in Supplemental Tables 1–3.

Photochemical activation kinetics were measured to assess the efficiency of the newly designed photoreactor by irradiation of a stock solution of DFO-PEG_3_-ArN_3_. Samples were analyzed by reverse-phase high-performance liquid chromatography (HPLC). The irreversible photoactivation of DFO-PEG_3_-ArN_3_ was complete within 90 s, as indicated by full consumption of the starting material in HPLC (Fig. 3b).

The three model reactions were used to assess different aspects of the automated system. First, the photoactivatable fluorophore RhodB-PEG_3_-ArN_3_ was used to optimize the sequence and timing of the different steps in the automation protocol. The use of a highly colored (pink) RhodB-PEG_3_-ArN_3_ dye also helped to evaluate the completeness of all reagent transfers by visual tracking and by quantitative measurements of dilution factors using electronic absorption spectroscopy. We discovered that the lipophilic nature of the small-molecule RhodB-PEG_3_-ArN_3_ compound, and the related photolyzed products, means that these species stick to Sephadex columns which are typically used for protein purification. Consequently, the fluorescently labeled RhodB-PEG_3_-azepin-HSA product can be purified easily from the small-molecule byproducts on the ALISI system by using standard PD-10 columns but this model fluorophore was not ideal for optimizing the purification procedure for radiolabeled proteins.

Next, test reactions between HSA and pre-radiolabeled ^68^GaDFO-PEG_3_-ArN_3_ were used to optimize the photochemical conjugation step. Optimization was performed by maximizing the decay-corrected radiochemical conversion (RCC, measure by size-exclusion chromatography high-performance liquid chromatography, SEC-HPLC) to the labeled protein, ^68^GaDFO-PEG_3_-azepin-HSA. We found that pH is a critical factor in determining the yield of photo-induced bioconjugation reactions using ArN_3_ species^27^. Therefore, we investigated the use of different buffers with the aim of controlling pH throughout all synthetic steps. 2-[4-(2-hydroxyethyl)piperazin-1-yl]ethanesulfonic acid (HEPES) is a zwitterionic buffer commonly used in the synthesis or radiolabeled mAbs^17^. In our hands, the use of HEPES at concentrations ≥ 0.5 M had a detrimental impact on the RCC of ^68^GaDFO-PEG_3_-azepin-HSA (data not shown). In contrast, reactions in sodium borate buffer (0.25 M, pH8) did not quench the light-induced conjugation reaction. Experimental data showing the measured RCC *versus* reaction pH are presented in Fig. 3c. These data show a trend toward increased RCC in the pH window from 8.0-to-9.5, with RCC reaching 47% between pH8.0–8.7. Below pH8.0, conjugation yields decrease sharply due to progressive protonation of lysine residues. Based on these data, further reaction parameters were optimized at pH8.0.

Our goal was to minimize the manual handling of radioactive components by producing ^89^Zr-mAbs directly from ^89^Zr-oxalate stocks and the unfunctionalized protein. Therefore, we examined the complexation reaction to form ^89^ZrDFO-PEG_3_-ArN_3_ on ALISI (Fig. 3d)^24,25^. ^89^Zr-oxalate was transferred into the reaction vial, and then neutralized by automatic transfer of the Na_2_CO_3_ and sodium borate solutions. Then, the DFO-PEG_3_-ArN_3_ solution was transferred into the magnetically stirred vial, and 10 s after addition, an aliquot of the reaction mixture was removed manually with a pipette and spotted immediately onto a silica gel instant-thin-layer chromatography (ITLC) strip (developed with 50 mM DTPA, pH7.4). The radio-ITLC data showed quantitative radiochemical conversion to give ^89^ZrDFO-PEG_3_-ArN_3_ (which is retained at the baseline, *R*_f_ = 0.0) in < 10 s. In contrast to radiolabeling of pre-functionalized DFO-mAbs which requires > 45 min, the high diffusion coefficient of the small-molecule components and gentle stirring of the reaction mixture facilitate rapid ^89^Zr-complexation by DFO-PEG_3_-ArN_3_.

Data in Fig. 3e show the effect of changing the protein concentration on the measured photochemical conversion yields (PCY) for the synthesis of RhodB-PEG_3_-azepin-HSA (purple), or RCC yields for ^68^GaDFO-PEG_3_-azepin-HSA (blue) and ^89^ZrDFO-PEG_3_-azepin-HSA (black). As with many bioconjugation reactions, photo-induced labeling using ArN_3_ compounds show a steep dependence on the initial concentration of protein. Conjugation yields began to decrease when [HSA] < 10 mg mL^-1^. For each of the RhodB-PEG_3_-azepin-HSA (purple trace), ^68^GaDFO-PEG_3_-azepin-HSA (blue), and ^89^ZrDFO-PEG_3_-azepin-HSA products, maximum conjugation yields were obtained at [HSA] ~ 15 mg mL^-1^ with values of 34.2 ± 0.9%, 49.1 ± 3.9% and 62.9 ± 2.2%, respectively. Analysis by size-exclusion chromatography coupled to a high-performance liquid chromatography system (SEC-HPLC, Fig. 3f) confirmed the successful protein-ligation by co-elution of the HSA protein (orange trace) with that of RhodB-PEG_3_-azepin-HSA (purple trace, monitoring the fluorophore at 568 nm), and the radioactive traces of ^68^GaDFO-PEG_3_-azepin-HSA (blue trace), and ^89^ZrDFO-PEG_3_-azepin-HSA (black trace).

**Figure 4:**
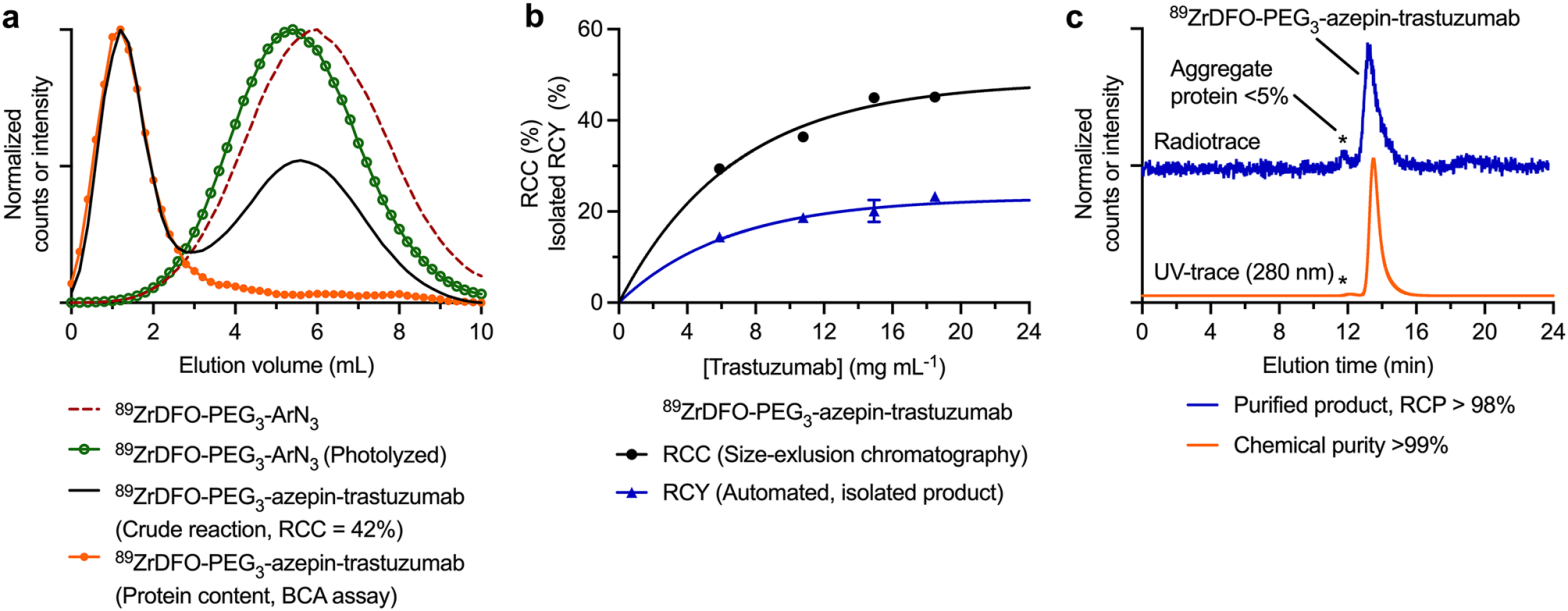
Chromatographic data on the photoradiosynthesis of ^89^Zr-labeled proteins. (**a**) SK-10 size-exclusion elution profiles of ^89^ZrDFO-PEG_3_-ArN_3_ (dashed-red), the photolyzed solution of ^89^ZrDFO-PEG_3_-ArN_3_ in the absence of protein (green), the crude reaction containing the ^89^ZrDFO-PEG_3_-azepin-trastuzumab product (black), and the measured protein content in the crude reaction mixture (orange). (**b**) Experimental data on the radiochemical conversion (RCC/%; black; measured after the photolysis step) and the decay-corrected isolated radiochemical yields (RCY/%; blue) for the automated photoradiosynthesis of ^89^ZrDFO-PEG_3_-azepin-trastuzumab using ALISI with different initial concentrations of trastuzumab. (**c**) SEC chromatograms of the isolated ^89^ZrDFO-PEG_3_-azepin-trastuzumab product (produced by full automation using ALISI) showing the radioactive profile (blue) and the protein elution profile measured by electronic absorption at 280 nm (orange).

The purification of ^89^Zr-mAbs typically involves using PD-10 desalting columns (Sephadex G-25, 5 kDa exclusion limit) which only work well when very high RCCs are obtained, or when the small-molecule byproducts are trapped on the Sephadex (as with the RhodB-PEG_3_-azepin-HSA synthesis, vide supra). However, when RCCs drop below ~ 80%, the separation efficiency of the PD-10 matrix is sub-optimal. To enhance the radiochemical purity (RCP) of the purified ^89^Zr-labeled proteins, we constructed custom-made SK-10 columns that have the same geometry as PD-10 columns but are loaded with Sephadex® G-100 (150 kDa exclusion limit) that increases the resolving power. SK-10 columns had a dead volume of ~ 2 mL followed by a protein collection volume of 2.5 mL (Fig. 4a). Small-molecules were effectively retained on SK-10 columns, where a peak-to-peak separation between the desired ^89^ZrDFO-PEG_3_-azepin-trastuzumab and the byproducts was > 3 mL, giving a final RCP of the purified protein fraction of > 99% starting from a crude reaction mixture that had an RCC of 42%.

Next, we investigated the fully automated production of ^89^ZrDFO-PEG_3_-azepin-trastuzumab using ALISI (Supplemental Table 4). Again, changing the protein concentration had an impact on both decay-corrected RCC for the protein conjugation step and on the isolated decay-corrected RCY of ^89^ZrDFO-PEG_3_-azepin-trastuzumab where a plateau was observed in the protein concentration range of 10–15 mg mL^-1^ (Fig. 4b). The same trend observed in reactions with HSA was seen with Herceptin. Automation successfully gave purified ^89^ZrDFO-PEG_3_-azepin-trastuzumab in < 25 min starting directly from non-purified Herceptin (14.9 mg mL^-1^ of protein) with an isolated decay-corrected RCY of 20.1 ± 2.4% (*n* = 3), and an RCP > 99% (Fig. 4c). Notably, SEC analysis of the isolated ^89^ZrDFO-PEG_3_-azepin-trastuzumab product revealed less than 5% of the activity was associated with an aggregated protein fraction (which elutes at a slightly shorter retention time). The activity recovered in the purified product was lower than the RCC by a factor of ~ 2. By tracking the activity along the fluid pathway, we found that ^89^Zr-oxalate was loaded into the reaction vial with 82.7 ± 3.1% efficiency. The photochemical conjugation efficiency is given by the RCC (~ 45%) and loading of the crude reaction mixture onto the SK-10 column had a transfer efficiency > 99%. The purification step was, on average, 75.0 ± 4.3% efficient, while sterile filtration led to a small loss in protein-bound activity with a recovered activity efficiency of 87.7 ± 2.8%. Transfer losses account for the difference between the measured RCC and RCY values.

Qualitative testing of the ^89^ZrDFO-PEG_3_-azepin-trastuzumab products for residual borate was performed by adding a few drops of an ethanolic solution of curcumin, which produces a red/orange solution due to the formation of rosocyanine dye in the presence of borate species^28^. Visual detection is possible at borate concentrations down to ~ 2.5 mM and tests confirmed that none of the isolated samples of ^89^ZrDFO-PEG_3_-azepin-trastuzumab produced by ALISI contained residual sodium borate.

In the clinic, patients undergoing PET scans with ^89^Zr-mAbs typically receive a dose of 37 MBq of activity administered with between 3 – 100 mg of total protein^29–31^. In scaled-up syntheses (Supplemental Table S4), we demonstrated the potential of ALISI to produce individual patient doses of ^89^ZrDFO-PEG_3_-azepin-trastuzumab. Starting from 152 MBq of ^89^Zr-oxalate and 9.7 mg of trastuzumab (formulated as Herceptin), photoradiosynthesis gave an activity yield of 27.9 MBq of isolated ^89^ZrDFO-PEG_3_-azepin-trastuzumab with a decay corrected RCY of 18.3%, an RCP > 99%, a chemical purity > 99%, and a molar activity *A*_m_ of 0.43 MBq nmol^-1^ of protein. If the ALISI system was qualified for use in a clinical radiopharmacy, this reaction product would be sufficient to image a patient.

**Figure 5:**
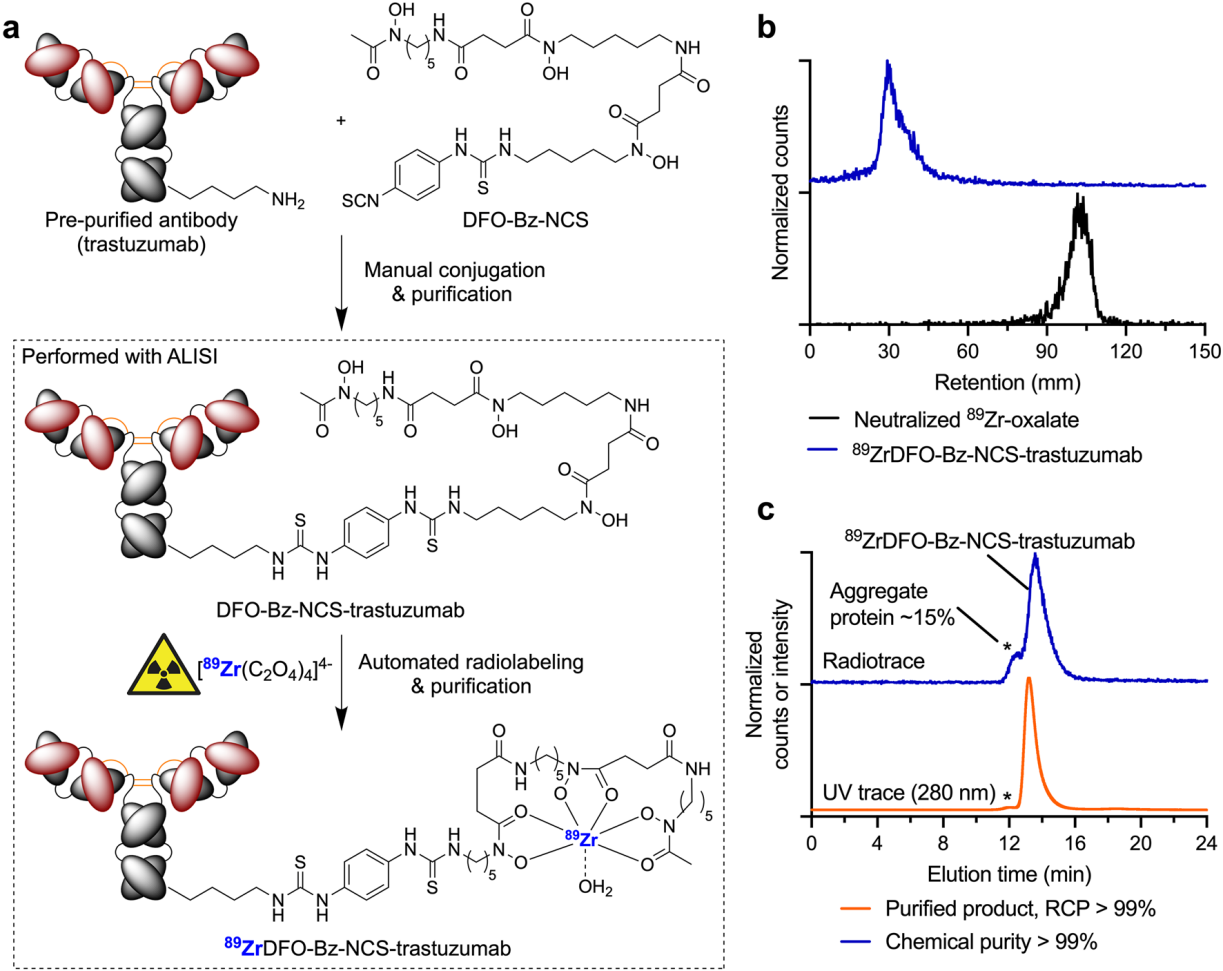
Automated radiosynthesis and purification of conventional ^89^Zr-mAbs using ALISI. (**a**) Reaction scheme showing the two-step bioconjugation and ^89^Zr-radiolabeling of trastuzumab^16,17^. (**b**) Radio-ITLC traces (DTPA eluent) of ^89^ZrDFO-Bz-NCS-trastuzumab (blue, *R*_f_ = 0.0) and the control showing neutralized ^89^Zr-oxalate (black, *R*_f_ = 1.0). (**c**) SEC chromatograms of ^89^ZrDFO-Bz-NCS-trastuzumab product produced by full automation using ALISI) showing the radioactive trace (blue) and the protein elution profile measured by electronic absorption at 280 nm (orange).

Finally, to illustrate the flexibility of the ALISI radiosynthesizer, we adapted the system to automate the radiolabeling and purification of ^89^Zr-mAbs via the conventional two-step approach (Fig. 5a). First, trastuzumab was recovered as a purified protein from a clinical-grade sample of formulated Herceptin by using PD-10 gel filtration. Next, the functionalized DFO-Bz-NCS-trastuzumab radiolabeling precursor was produced in accordance with the methods of Vosjan et al.^16,17^. Finally, ^89^Zr-radiolabeling and automated PD-10 purification using ALISI gave ^89^ZrDFO-Bz-NCS-trastuzumab with a decay-corrected isolated RCY of 48.3 ± 8.4% (*n* = 3), an RCP > 99%, and chemical purity > 99% (Supplemental Table S5) in 90 min. Corresponding radio-ITLC and SEC-HPLC characterization data for ^89^ZrDFO-Bz-NCS-trastuzumab are shown in Fig. 5b and c, respectively. Importantly, the purified sample of ^89^ZrDFO-Bz-NCS-trastuzumab contained ~ 15% of ^89^Zr-activity associated with an aggregated protein peak. This is common feature of the conventional two-step radiolabeling method used in the clinic, which requires removal of the mAb from the stabilizing formulation components prior to functionalization with DFO-Bz-NCS^23^. The presence of ^89^Zr-labeled protein aggregate is associated with accumulation of activity in the liver and spleen. In comparison, the photoradiochemical approach reduces this aggregate fraction by a factor of ~ 3 which would likely improve image contrast and reduce the radiation burden to the patient.

## Discussion

The ALISI radiosynthesizer was constructed by using a combination of open-source microcontrollers (Arduino) and computer-aided design (CAD), coupled with additive manufacturing^32,33^. Arduino electronic components are modular and expandable. This allows for rapid and facile integration of new features during prototype development. Liquid handling components and all fluidic pathways employ commercially available, single-use, sterile tubing and three-way switching valves operated by digital servomotors. These components were chosen to facilitate future translation of ALISI to a clinical environment. The custom-made, electropolished photoreactor houses three high-powered LEDs with peak emission at 365 nm (Fig. 2c). Light-induced activation of our ArN_3_ reagents is rate-limiting in the photoradiosynthesis of ^89^Zr-mAbs, and in comparison with manual reactions, the mirrored photoreactor of ALISI gave a 5-to-10-fold decrease in reaction time^26^.

It is important to note that the bioconjugation efficiency (as measured from the PCY or RCC values) shows a strong dependence on the reaction geometry and the conditions used. For instance, the nature of the photoactive compound and protein substrate, the choice of radionuclide, the concentrations of all reagents, the buffer composition, and the pH influence conjugation efficiency. Under identical conditions, RhodB-PEG_3_-ArN_3_ gave consistently lower yields for HSA labeling than pre-radiolabeled ^68^GaDFO-PEG_3_-ArN_3_, which in-turn, was lower than radiolabeling with ^89^ZrDFO-PEG_3_-ArN_3_ generated in situ. Under our optimized conditions, the observed bioconjugation yields using an initial 1:1 stoichiometric ratio between HSA and the photoactive compound were in the range of 35%–65%. Standard bioconjugation reactions used to make the DFO-mAbs, including activated ester^16^ or thiourea^17^ chemistry, have efficiencies in the range of ~ 20%–75% (see Supplementary Methods). These data confirm that photo-induced bioconjugation is equally successful compared to current state-of-the-art methods used to make radiolabeled mAbs in clinical practice. For the photoradiosynthesis of ^89^ZrDFO-PEG_3_-azepin-trastuzumab from Herceptin, the RCC was ~ 45%. This is remarkable considering that the radiolabeling and bioconjugation steps are complete in < 90 s, and the reaction uses non-purified mAb where the mixture contains all formulation components of clinical-grade Herceptin. We note that radiochemists performing manual syntheses of ^89^Zr-mAbs are familiar with obtaining near quantitative RCYs, but in most cases, the reaction only involves the radiolabeling step (not the conjugation). Since test reactions are usually employed to predetermine the maximum molar activity of a sample, quantitative labeling is expected. In a recent study, Poot et al. automated the radiolabeling and purification of ^89^Zr-mAbs from pre-functionalized DFO-mAbs with RCYs in the range 60%–75%^3^. With the present photochemistry, formation of ^89^ZrDFO-PEG_3_-ArN_3_ in situ is quantitative and the decay-corrected isolated RCY of ^89^ZrDFO-PEG_3_-azepin-trasutzumab (20.1 ± 2.4%) encompasses all chemical reactions, transfers, and processing steps. Transfer losses are one of the main limitations of adapting manual chemistry to automated platforms. Considering the multi-step nature of the ALISI protocol, which includes all reagent transfers, acid neutralization, buffer control, radiolabeling and bioconjugation, in-line purification, sterile filtration, and product formulation, the observed RCYs are an excellent benchmark.

Purification using SK-10 columns filled with Sephadex® G-100 media provided greatly enhanced separation and improved RCP (> 99%) of the isolated ^89^ZrDFO-PEG_3_-azepin-trasutzumab when compared with PD-10 columns. This is expected since the Sephadex G-25 media used in PD-10 columns is primarily intended for desalting and is sub-optimal for separating large proteins from small-molecule components.

Finally, automated radiosynthesis of ^89^Zr-mAbs on ALISI improved the reproducibility of the chemistry by standardizing the reaction geometry and conditions, and by decreasing the potential for user-related, irreproducible errors. Collectively, the successful synthesis and isolation of ^89^ZrDFO-PEG_3_-azepin-trastuzumab using our automated radiosynthesizer suggest that ALISI is potentially useful for preparing individual patient doses or small batches of ^89^Zr-mAbs (and other radiolabeled proteins) on demand. With further development, we anticipate that the system can be adapted for use with other radionuclides and photoactivatable chelates to access radiopharmaceuticals for applications in radioimmunotherapy.

## Conclusion

We developed a radiosynthesizer unit that performs the fully automated light-induced synthesis of ^89^Zr-mAbs for immunoPET. The ALISI system can produce ^89^Zr-mAbs in < 25 min in high radiochemical purity (RCP > 99%) starting from stock solutions of ^89^Zr-oxalate, a photoactivatable DFO-PEG_3_-ArN_3_ chelate, and a protein of interest. Features of the automated protocol include neutralization of the oxalic acid, buffer exchange and pH correction, quantitative formation of the photoactivatable ^89^ZrDFO-PEG_3_-ArN_3_ complex, rapid (< 90 s) light-induced protein-ligation, in-line purification using novel SK-10 size-exclusion chromatography columns, and finally sterile filtration and product formulation in a biocompatible medium. After loading the reagent reservoirs, all steps are performed at the touch of a single button. The system is also highly flexible, producing purified ^89^Zr-mAbs via conventional labeling of a pre-functionalized DFO-mAb conjugate. With further development, we anticipate that ALISI could facilitate on-demand access to individual patient doses of ^89^Zr-mAbs in Nuclear Medicine facilities that rely on automated devices for radiotracer production.

## Methods

Further details are presented in the Supplemental Materials. Schematics and sample code for operating the microcontrollers are available on request.

### Protein samples

Formulated Herceptin™ (Roche/Genentech, South San Francisco, CA) and human serum albumin (HSA; Merck, Darmstadt, Germany) were reconstituted in water (> 18.2MΩ·cm at 25 °C). Protein concentrations were determined by using a Nanodrop™ One^C^ Microvolume UV–Vis Spectrophotometer.

### Additive manufacturing

The ALISI radiosynthesizer was constructed by using the computer assisted design (CAD) software Solidworks2020 (Dassault Systèmes, Vélizy-Villacoublay, France). Components were prepared through additive manufacturing, laser cutting or purchased directly from commercial vendors. Additive manufacturing components were produced by selective laser sintering and made from PA2200, a fine powder based on polyamide-12. The synthesizer case was manufactured from laser cut, 5 mm plates of high tensile strength, black polyoxymethylene (POM) and an aluminum profile modular assembly system (Kanya AG, Rüti, Switzerland).

### Control and electronics

Electronic components are based around the single-chip ATmega328 microcontroller. Microcontrollers, breakout boards, additional circuit boards, and electronic components were either custom-manufactured or purchased from Arduino.cc (Ivrea, Italy), Adafruit Industries (New York City, NY, USA), RS Components (Frankfurt-am-Main, Deutschland), or Distrelec AG (Nänikon, Switzerland). The photoreactor consists of an electropolished stainless steel tube containing an array of three, high-powered light-emitting diodes (LEDs; Nichia, Anan, Japan) with a light output of 1.03 W per LED at 365 nm. Valve modulation is performed with standard digital servos (Savöx, Salt Lake City, UT, USA). All liquid transfer steps are driven pneumatically with a syringe pump controlled by a NEMA-17 bipolar stepper motor (Distrelec AG, Nänikon, Switzerland).

### Liquid handling and cassettes

All components for the disposable cassette system were either custom-manufactured or purchased from commercial vendors (B. Braun Melsungen AG, Meslungen, Germany, or BD, Heidelberg, Germany). The cassettes use flexible, high chemical resistance Tygon tubing. For purification, custom-made SK-10 separation columns (Econo-Pac chromatography columns, Bio-Rad Laboratories, Hercules, CA, USA) filled with 8.3 mL of pre-soaked Sephadex® G-100 (Merck, Darmstadt, Germany) were constructed. The stationary phase of the SK-10 columns was capped with filter-frits and columns were eluted with sterile PBS (pH7.4).

### Synthesis and radiochemistry

The photoactivatable chelate DFO-PEG_3_-ArN_3_ and the fluorescent photoactivatable fluorophore (*PhotoTag*) RhodB-PEG_3_-ArN_3_ were synthesized and characterized as described previously^24^. The stock solution of ^89^Zr-oxalate ([^89^Zr(C_2_O_4_)_4_]^4-^(aq.) in ~ 1 M oxalic acid) was obtained from PerkinElmer (Waltham, MA, USA; manufactured by the BV Cyclotron VU, Amsterdam, The Netherlands) and was used without further purification. Full experimental details on the model chemistry using RhodB-PEG_3_-ArN_3_, ^68^GaDFO-PEG_3_-ArN_3_ and ^89^Zr-oxalate in combination with DFO-PEG_3_-ArN_3_ are given in the Supplemental Information. All reported RCC and RCY values are decay corrected.

### Setup for automated radiolabeling

Reactions with ALISI were performed on new liquid handling equipment, freshly assembled from sterile packaging. Briefly, individual reservoirs were assigned to stock solutions of Na_2_CO_3_(aq.), DFO-PEG_3_-ArN_3_, ^89^Zr-oxalate, protein, reaction buffer, and sterile PBS for purification by size-exclusion chromatography (SEC) (see Supplemental Tables 1–4). A minimum volume of ~ 50μL was necessary to achieve adequate liquid transfer. For test reactions where reagent volumes were below this threshold, the total volume was adjusted with an appropriate volume of water.

### Description of the automated ^89^Zr-photoradiolabeling procedure

A schematic of the plumbing diagram and a photograph of the ALISI system set-up for the synthesis of ^89^Zr-mAbs is shown in Fig. 2. The radiosynthesizer unit is initialized by pushing the power button. After initialization, the system provides a digital prompt on the built-in LCD-display to indicate that the device is ready to start. Automated radiosynthesis and purification is initiated by pressing the start button. Thereafter, the radiosynthesizer transfers all reagents and components to the reaction vial, located inside the photoreactor, in the following sequence: *i*) ^89^Zr-oxalate in ~ 1 M oxalic acid is transferred. *ii*) An equal volume of 1 M Na_2_CO_3_(aq.) is transferred. *iii*) DFO-PEG_3_-ArN_3_ in a solution of ~ 10% DMSO and aqueous sodium borate buffer (0.25 M, pH8.0) is removed from the reservoir and first used to wash the ^89^Zr-stock solution reservoir before being delivered to the reaction vial. *iv*) The protein solution is transferred. *v*) Additional sodium borate buffer (0.25 M, pH8.0) is used to wash the protein reservoir and then transferred.

After the reagent transfer sequence, the reaction mixture is irradiated with 365 nm light for 90 s. Tests indicated that the temperature of the reaction mixture does not change during this time, but the high-powered LEDs require cooling with an aluminum heat-sink attached to a fan. After irradiation, the crude mixture is transferred to the SK-10 size-exclusion column (SEC) for purification. After automatic separation, the product fraction containing the high molecular weight protein is filtered through a standard 0.22 μm sterile filter and collected in a sterile vial. The product is formulated in sterile PBS (pH7.4), and after quality control, is ready for use in radiochemical, cellular, or in vivo assays.

### Data analysis

Data were plotted by using the GraphPad Prism 9.0 software (GraphPad Software Inc., San Diego, California USA).

## Supplementary Information

Supplementary Information.

## Data Availability

An electronic computer-aided design file containing the full assembly and all components of the radiosynthesizer required for 3D-printing, as well as the Arduino program (sketch) is available from the corresponding author.
