# Supplementary material for: Automated light-induced synthesis of 89Zr-radiolabeled antibodies for immuno-positron emission tomography
Source: Sci Rep. 2022 Jan 13;12:668. doi: 10.1038/s41598-021-04626-5 (PMC8758695; doi:10.1038/s41598-021-04626-5)
Supplement: Supplementary file 1 — Supplementary Information. [file 41598_2021_4626_MOESM1_ESM.pdf]

Automated Light induced Synthesis of ^{89}Zr-Radiolabeled Antibodies for Immuno-Positron Emission Tomography

Simon Klingler[1] and Jason P. Holland[1]*

[1] University of Zurich, Department of Chemistry, Winterthurerstrasse 190, CH-8057, Zurich, Switzerland

* **Corresponding Author:**

Prof. Dr Jason P. Holland

Department of Chemistry

University of Zurich

Winterthurerstrasse 190

CH-8057, Zurich, Switzerland

Tel: +41-44-63-53990

E-mail: jason.holland@chem.uzh.ch

Website: www.hollandlab.org

Twitter: @HollandLab_

orcid.org/0000-0002-0066-219X

First-author:

Simon Klingler, E-mail: simon.klingler@chem.uzh.ch

orcid.org/0000-0001-9951-1609

Table of contents

Materials and Methods 3

Model reactions with the RhodB-PEG_3-ArN_3 4

Automated reactions 4

Radiochemistry 5

Automated reactions with pre-synthesized ^{68}GaDFO-PEG_3-ArN_3 5

Optimization of ^{89}Zr photoradiosynthesis of protein conjugates with HSA 5

Automated photoradiosynthesis of ^{89}ZrDFO-PEG_3-azepin-trastuzumab 5

Preparation of DFO-Bz-NCS-trastuzumab 5

Chelate number determination of DFO-Bz-NCS-trastuzumab 5

Automated ^{89}Zr-radiolabelling of DFO-Bz-NCS-trastuzumab 6

Supplemental Table 1. Reagents and conditions used for reactions with RhodB-PEG_3-ArN_3 and HSA. 7

Supplemental Table 2. Reagents and conditions used for reactions with pre-labeled ^{68}GaDFO-PEG_3-ArN_3 and HSA. 8

Supplemental Table 3. Reagents and conditions used for reactions with ^{89}Zr, DFO-PEG_3-ArN_3 and HSA. 9

Supplemental Table 4. Reagents and conditions used for reactions with ^{89}Zr, DFO-PEG_3-ArN_3 and Herceptin™ 10

Supplemental Table 5. Reagents and conditions used automated radiolabeling of DFO-Bz-NCS-trastuzumab with ^{89}Zr. 11

References 12

Materials and Methods

Unless otherwise stated, all chemicals were of reagent grade and purchased from SigmaAldrich (St. Louis, MO), Merck (Darmstadt, Germany), Tokyo Chemical Industry (Eschborn, Germany), abcr (Karlsruhe, Germany) or CheMatech (Dijon, France). Water (>18.2 MΩ·cm at 25 °C, Puranity TU 3 UV/UF, VWR International, Leuven, Belgium) was used without further purification. Solvents for reactions were of reagent grade, and where necessary, were dried over molecular sieves. Evaporation of the solvents was performed under reduced pressure by using a rotary evaporator (Rotavapor R-300, Büchi Labortechnik AG, Flawil, Switzerland) at the specified temperature and pressure. Electronic absorption spectra were recorded using a Nanodrop™ One^{C} Microvolume UV-Vis Spectrophotometer (ThermoFisher Scientific, supplied by Witec AG, Sursee, Switzerland). Protein concentration was determined in accordance with the manufacturers protocol.

[^{68}Ga][Ga(H_2O)$_6$]Cl_3(aq.) was obtained from ^{68}Ge/^{68}Ga-generators (Eckert&Ziegler, Model IGG100 Gallium-68 Generator), eluted with 0.1 M HCl(aq.). The eluted ^{68}Ga activity was trapped and purified by using a strong cation exchange column (Strata-XC, [SCX], Eckert&Ziegler). [^{68}Ga][Ga(H_2O)$_6$]Cl_3(aq.) was eluted from the SCX cartridge by using a solution containing 0.13 M HCl(aq.) and approx. 5 M NaCl(aq.) (SCX eluent).

For radiolabeling experiments the ^{68}Ga stock solution or ^{89}Zr-oxalate stock solution were typically added as the limiting reagent. Radioactive reactions were monitored by using instant thin-layer chromatography (iTLC). Glass-fibre iTLC plates impregnated with silica-gel (iTLC-SG, Agilent Technologies) were developed in citrate buffer (pH = 4.5, 0.2 M) for ^{68}Ga or DTPA (50 mM, pH = 7.2) for ^{89}Zr and analyzed on a radio-TLC detector (SCAN-RAM, LabLogic Systems Ltd, Sheffield, United Kingdom). Radiochemical conversion (RCC) was determined by integrating the data obtained by the radio-TLC plate reader and determining both the percentage of radiolabelled product (R_f = 0.0) and 'free' ^{68}Ga or ^{89}Zr (R_f = 1.0; present in the analyses as [^{68}Ga]Ga-citrate or [^{89}Zr]Zr-DTPA). Integration and data analysis were performed by using the software Laura version 5.0.4.29 (LabLogic,) and all measurement include appropriate background and decay corrections.

DFO-PEG$_3$-ArN_3 and RhodB-PEG$_3$-ArN_3 were prepared in accordance with our previously published procedures.(*1*) Measurements of the photodegradation kinetics were performed in accordance with previously published methods that were adapted for use on the ALISI box. Photodegradation reactions with DFO-PEG$_3$-ArN_3 were performed in the reaction buffer.(2) Irradiation was performed in 10 second intervals, after which, aliquots of the reaction

mixture were removed and samples were analyzed by UHPLC. The experiment was performed in duplicate. Using the ALISI system, photoactivation of the aryl azide group on DFO-PEG_3-ArN_3 was essentially complete (>95% conversion) after only 90 s. Data from the integration of the peak corresponding to the starting material in the UHPLC chromatograms were normalized to the initial peak area (from the trace recorded at t = 0 s) and fitted with a standard first-order exponential decay.

^{68}GaDFO-PEG_3-ArN_3 was typically prepared through the addition of a solution of DFO-PEG_3-ArN_3 in DMSO to the ^{68}Ga stock in SCX eluent. The pH of the solution was quickly adjusted to pH ~4 by the addition of 1 M $NaHCO_3$(aq.). The complexation reaction between $^{68}Ga^{3+}$ ions and the DFO chelate is immediate and reactions were complete in <10 s.

$^{89}Zr(C_2O_4)_4]^{4-}$ (^{89}Zr-oxalate) was obtained as a solution in approximately 1.0 M oxalic acid from PerkinElmer (Waltham, MA, USA; manufactured by the BV Cyclotron VU, Amsterdam, The Netherlands) and was used without further purification.

^{89}Zr-DFO-PEG_3-ArN_3 can be prepared from ^{89}Zr-oxalate either by neutralization with 1 M Na_2CO_3(aq.) followed by the addition of DFO-PEG_3-ArN_3 (0.4 mM in DMSO), or by adding ^{89}Zr-oxalate to as solution of DFO-PEG_3-ArN_3 (0.4 mM in DMSO) in sodium borate buffer (0.25 M, pH 8). The complexation reaction between $^{89}Zr^{4+}$ ions and the DFO chelate is immediate and reactions were complete in <10 s.

Model reactions with the RhodB-PEG_3-ArN_3

Automated reactions

For the model reactions with RhodB-PEG_3-ArN_3, the ALISI radiosynthesizer was loaded as follows: the reservoir usually used for Na_2CO_3 was filled with a solution of H_2O, the reservoir for the DFO-PEG_3-ArN_3 in buffer solution was replaced with only buffer solution, and the reservoir for the ^{89}Zr-stock solution was filled with RhodB-PEG_3-ArN_3 (10 µL of 10 mM in DMSO) and H_2O (40 µL). Reaction conditions are given in Table S1 (below). Reactions were performed duplicate.

Radiochemistry

Automated reactions with pre-synthesized ^{68}GaDFO-PEG$_3$-ArN$_3$

For model reactions with ^{68}Ga the reservoir usually used for Na_2CO_3 is filled with a solution of H_2O, the reservoir for the DFO-PEG$_3$-ArN$_3$ in buffer solution was substituted with only buffer solution, and the reservoir for the ^{89}Zr-stock solution is filled with freshly prepared ^{68}GaDFO-PEG$_3$-ArN$_3$. Reaction conditions are given in Table S2 (below). Reactions were performed duplicate.

Optimization of ^{89}Zr photoradiosynthesis of protein conjugates with HSA

For the reactions with ^{89}Zr, the reservoirs were loaded in accordance with the plumbing diagram shown in the main manuscript (Figure 2). Reaction conditions are given in Table S3 (below). Reactions were performed duplicate.

Automated photoradiosynthesis of ^{89}ZrDFO-PEG$_3$-azepin-trastuzumab

For the synthesis of ^{89}ZrDFO-PEG3-azepine-trastuzumab, the reservoirs were loaded in accordance with the plumbing diagram shown in the main manuscript (Figure 2). Reaction conditions are given in Table S4 (below).

Preparation of DFO-Bz-NCS-trastuzumab

DFO-Bz-NCS-trastuzumab was obtained by following the procedure of Vosjan *et al.*(*2*) Briefly, trastuzumab was pre-purified from a clinical grade sample of Herceptin™ (Genentech, CA, USA) by manual PD-10 SEC and recovered in saline. Functionalization was performed by using a 5-fold molar excess of commercially available *p*-NCS-Bz-DFO chelator (CheMatech, France) with respect to the moles of trastuzumab. Manual PD-10 SEC of the crude reaction mixture was performed to remove excess small molecule components and gave DFO-Bz-NCS-trastuzumab (9.9 mg, 5.0 mg mL^{-1}) in saline (pH7.4).

Chelate number determination of DFO-Bz-NCS-trastuzumab

The molar activity of the ^{89}Zr-oxalate stock solution was measured by isotopic dilution assays. Briefly, a stock solution of desferrioxamine B mesylate was prepared in water (3 mg mL^{-1}, [DFO] = 4.63 mM). A 1:10 dilution series of DFO was prepared in microcentrifuge tubes. Then

an aliquot of a neutralized [^{89}Zr][$Zr(C_2O_4)_4$]$^{-4}$ (aq.) stock solution was added to each tube (~1.5 MBq). Reactions were vortexed and incubated at room temperature for 90 min. to ensure complete reaction occurred. At the end of the reaction, aliquots were spotted onto iTLC plates and developed by using an aqueous mobile phase containing DTPA (50 mM, pH7.4). Radio-iTLC analysis was used to measure the radiochemical conversion (RCC) with the product [^{89}Zr]ZrDFO retained at the baseline (R_f = 0.0) and any 'free' Zr^{4+} ions, which are complexed as [^{89}Zr][Zr(DTPA)]$^{-}$, eluting at the solvent front (R_f = 1.0). The experimentally measured molar activity of the [^{89}Zr][$Zr(C_2O_4)_4$]$^{4-}$ (aq.) stock solution was A_m = 9.54 MBq nmol^{-1}.

The average number of accessible chelates per protein molecule of the DFO-Bz-NCS-trastuzumab sample was measured by determining the maximum molar activity of [^{89}Zr]ZrDFO-Bz-NCS-trastuzumab by radiolabeling with an excess of activity following published procedures.(*2*) Experimentally, the maximum molar activity of [^{89}Zr]ZrDFO-Bz-NCS-trastuzumab with respect to the moles of protein was determined to be 34.2 MBq nmol^{-1}. The chelate number is given by the ratio of the molar activity of the [^{89}Zr][$Zr(C_2O_4)_4$]$^{-4}$ (aq.) stock solution and maximum molar activity of [^{89}Zr]ZrDFO-Bz-NCS-trastuzumab and was determined to be 3.58 DFO chelates per antibody (which equates to an ~72% conjugation efficiency of the reaction between DFO-Bz-NCS and trastuzumab).

Automated ^{89}Zr-radiolabelling of DFO-Bz-NCS-trastuzumab

Radiolabeling of the DFO-Bz-NCS-trastuzumab was performed in HEPES buffer (0.5 M, pH 7.1–7.3) under conditions akin to published procedures.(*2*) For the reactions between ^{89}Zr and DFO-Bz-NCS-trastuzumab, the reservoirs were loaded in accordance with the plumbing diagram shown in the main manuscript (Figure 2) with the exception of the reservoir for the DFO-PEG$_3$-ArN$_3$, which was substituted for HEPES buffer (0.5 M, pH 7.1–7.3). Reaction conditions are given in Table S5 (below).

Supplemental Table 1. Reagents and conditions used for reactions with RhodB-PEG$_3$-ArN$_3$ and HSA.

Reservoir:	Reagent	A1 and A2	B1 and B2	C1 and C2	D1 and D2	E1 and E2	F1 and F2
Na_2CO_3	Na_2CO_3 (1 M) / µL	n/a[1]	n/a[1]	n/a[1]	n/a[1]	n/a[1]	n/a[1]
	H_2O / µL	50	50	50	50	50	50
DFO-PEG$_3$-ArN$_3$	DFO-PEG$_3$-ArN$_3$ (4 mM in DMSO) / µL	n/a	n/a	n/a	n/a	n/a	n/a
	Buffer (0.25 M, pH 8) / µL	100	100	100	100	100	100
PhotoTag	RhodB-PEG$_3$-ArN$_3$ (10 mM in DMSO) / µL	10	10	10	10	10	10
	H_2O/ µL	40	40	40	40	40	40
Protein	HSA (55.5 mg mL^{-1}) / µL	200	200	100	50	25	12.5
	Buffer (0.25 M, pH 8) / µL	0	0	100	150	175	187.5
Buffer	Buffer (0.25 M, pH 8) / µL	300	300	300	300	300	300
Total reaction volume / µL		700	700	700	700	700	700
Reaction protein content / mg		22.2	11.1	5.55	2.77	1.39	0.69
Reaction protein conc. / mg mL^{-1}		31.7	15.9	7.93	3.97	1.98	0.99
Results:	PCY (*n* = 2) / %	36.3 ± 3.1	34.2 ± 0.7	26.9 ± 1.0	19.3 ± 0.8	9.7 ± 0.0	7.6 ± 1.1

[1] n/a = not applicable

Supplemental Table 2. Reagents and conditions used for reactions with pre-labeled ^{68}GaDFO-PEG$_3$-ArN$_3$ and HSA.

All reactions were performed with 10-35 MBq of pre-radiolabeled ^{68}GaDFO-PEG-ArN$_3$

Reservoir:	Reagent	A1 and A2	B1 and B2	C1 and C2	D1 and D2
Na_2CO_3	Na_2CO_3 (1 M) / µL	n/a[1]	n/a[1]	n/a[1]	n/a[1]
	H_2O / µL	50	50	50	50
DFO-PEG$_3$-ArN$_3$	DFO-PEG$_3$-ArN$_3$ (4 mM in DMSO) / µL	n/a[1]	n/a[1]	n/a[1]	n/a[1]
	Buffer (0.25 M, pH 8) / µL	100	100	100	100
^{68}Ga	Pre-synthesized ^{68}GaDFO-PEG$_3$-ArN$_3$ / µL	50	50	50	50
	H_2O/ µL	0	0	0	0
Protein	HSA (55.5 mg mL^{-1}) / µL	200	100	50	25
	Buffer (0.25 M, pH 8) / µL	0	100	150	175
Buffer	Buffer (0.25 M, pH 8) / µL	300	300	300	300
Total reaction volume / µL		700	700	700	700
Reaction protein content / mg		11.1	5.55	2.77	1.39
Reaction protein conc. / mg mL^{-1}		15.9	7.93	3.97	1.98
Results:	RCC (*n* = 2) / % (measured by SEC)	49.1 ± 2.8	33.1 ± 0.3	25.6 ± 0.1	14.9 ± 0.0

[1] n/a = not applicable

Supplemental Table 3. Reagents and conditions used for reactions with ^{89}Zr, DFO-PEG$_3$-ArN$_3$ and HSA.

Note: * reactions were performed with 5-6 MBq of ^{89}Zr-oxalate the volume for both was adjusted ^{89}Zr-oxalate and Na_2CO_3 solution were adjusted accordingly. † Reactions A1 and A2 used a 1.5-fold higher HSA concentration (83.3 mg mL^{-1}).

Reservoir:	Reagent	A1 and A2	B1 and B2	C1 and C2	D1 and D2
Na_2CO_3	Na_2CO_3 (1 M) / µL	~5*	~5*	~5*	~5*
	H_2O / µL	~45	~45	~45	~45
DFO-PEG$_3$-ArN$_3$	DFO-PEG$_3$-ArN$_3$ (4 mM in DMSO) / µL	10	10	10	10
	Buffer (0.25 M, pH 8) / µL	90	90	90	90
^{89}Zr	^{89}Zr-oxalate stock (not neutralized) / µL	~5*	~5*	~5*	~5*
	H_2O/ µL	~45	~45	~45	~45
Protein	HSA (55.5 mg mL^{-1}) / µL †	200	200	100	50
	Buffer (0.25 M, pH 8) / µL	0	0	100	150
Buffer	Buffer (0.25 M, pH 8) / µL	300	300	300	300
Total reaction volume / µL		700	700	700	700
Reaction protein content / mg		16.6	11.1	5.55	2.77
Reaction protein conc. / mg mL^{-1}		23.8	15.9	7.93	3.97
Results:	RCC (n = 2) / % (measured by SEC)	63.1 ± 2.1	62.8 ± 1.6	45.5 ± 0.8	22.6 ± 3.0

Supplemental Table 4. Reagents and conditions used for reactions with ^{89}Zr, DFO-PEG$_3$-ArN$_3$ and Herceptin™

Note: * test reactions were performed with 5-6 MBq of ^{89}Zr-oxalate the volume for both ^{89}Zr-oxalate and Na_2CO_3 solution were adjusted accordingly. † The reaction was scaled up (Upscale 1) in activity and was performed with 30.9 MBq of ^{89}Zr-oxalate giving an activity yield of 5.8 MBq. ‡ The reaction was scaled up (Upscale 2) in activity and was performed with 152 MBq of ^{89}Zr-oxalate giving an activity yield of 27.9 MBq (equivalent to approximately 1 patient dose).

Reservoir:	Reagent	A	B	C	D	Upscale 1†	Upscale 2‡
Na_2CO_3	Na_2CO_3 (1 M) / µL	~5*	~5*	~5*	~5*	50	90
	H_2O / µL	~45	~45	~45	~45	0	0
DFO-PEG$_3$-ArN$_3$	DFO-PEG$_3$-ArN$_3$ (4 mM in DMSO) / µL	10	10	10	10	10	10
	Buffer (0.25 M, pH 8) / µL	140	140	140	140	140	110
^{89}Zr	^{89}Zr-oxalate stock (not neutralized) / µL	~5*	~5*	~5*	~5*	50	90
	H_2O/ µL	~45	~45	~45	~45	0	0
Protein	trastuzumab (64.7 mg mL^{-1}) / µL	200	150	100	50	150	150
	Buffer (0.25 M, pH 8) / µL	0	0	0	0	0	0
Buffer	Buffer (0.25 M, pH 8) / µL	250	250	250	250	250	200
Total reaction volume / µL		700	650	600	550	650	650
Reaction protein content / mg		12.9	9.71	6.47	3.24	9.70	9.70
Reaction protein conc. / mg mL^{-1}		18.4	14.9	10.7	5.88	14.9	14.9
Results:	RCC / % (measured by SEC)	45.1	45.0	36.4	29.4	n/a[1]	n/a[1]
	Decay-corrected RCY / %	23.3	22.8	18.7	14.4	19.1	18.4
	RCP / % (measured by SEC)	>99	>99	>99	>99	98	>99

[1] n/a = not applicable

Supplemental Table 5. Reagents and conditions used automated radiolabeling of DFO-Bz-NCS-trastuzumab with ^{89}Zr.

Note: reactions were performed with 9-11 MBq of ^{89}Zr-oxalate the volume for both was adjusted ^{89}Zr-oxalate and Na_2CO_3 solution were adjusted accordingly.

Reservoir:	Reagent	A	B	C
Na_2CO_3	Na_2CO_3 (1 M) / µL	8	8	8
	H_2O / µL	42	42	42
DFO-PEG$_3$-ArN$_3$	HEPES buffer (0.5 M, pH 7.1–7.3)/ µL	200	200	200
^{89}Zr	^{89}Zr-oxalate stock (not neutralized) / µL	8	8	8
	H_2O/ µL	42	42	42
Protein	DFO-Bz-NCS-trastuzumab (5.0 mg mL^{-1}mg mL^{-1}) / µL	200	200	200
Buffer	Buffer (0.25 M, pH 8) / µL	200	200	200
Total reaction volume / µL		700	700	700
Reaction protein content / mg		1	1	1
Reaction protein conc. / mg mL^{-1}		1.4	1.4	1.4
Results:	Decay-corrected RCY / %	48.8	39.7	56.5
	RCP / % (measured by SEC)	>99	>99	>99

[1] n/a = not applicable

References

1. Guillou A, Earley DF, Patra M, Holland JP. Light-induced synthesis of protein conjugates and its application in photoradiosynthesis of ^{89}Zr-radiolabeled monoclonal antibodies. *Nat Protoc*. 2020;15:3579-3594.

2. Vosjan MJWD, Perk LR, Visser GWM, et al. Conjugation and radiolabeling of monoclonal antibodies with zirconium-89 for PET imaging using the bifunctional chelate p -isothiocyanatobenzyl- desferrioxamine. *Nat Protoc*. 2010;5:739-743.
